# Design of Virtual Hands for Natural Interaction in the Metaverse

**DOI:** 10.3390/s24030741

**Published:** 2024-01-23

**Authors:** Joaquín Cerdá-Boluda, Marta C. Mora, Nuria Lloret, Stefano Scarani, Jorge Sastre

**Affiliations:** 1 Instituto de Instrumentación para Imagen Molecular (I3M), Universitat Politècnica de València, 46020 Valencia, Spain; 2 Departament d’Enginyeria Mecànica i Construcció (EMC), Universitat Jaume I, 12071 Castelló de la Plana, Spain; mmora@emc.uji.es; 3 Institute of Design and Manufacturing, Universitat Politècnica de València, 46022 Valencia, Spain; nlloret@upvnet.upv.es; 4 Department of Sculpture, Universitat Politècnica de València, 46022 Valencia, Spain; stesca2@upvnet.upv.es; 5 Institute of Telecommunications and Multimedia Applications, Universitat Politècnica de València, 46022 Valencia, Spain; jsastrem@upv.es

**Keywords:** AR, hand tracking, human–machine interaction, infrared sensor, markerless motion tracking, NFT, retopology, VR, WebXR, XR

## Abstract

The emergence of the Metaverse is raising important questions in the field of human–machine interaction that must be addressed for a successful implementation of the new paradigm. Therefore, the exploration and integration of both technology and human interaction within this new framework are needed. This paper describes an innovative and technically viable proposal for virtual shopping in the fashion field. Virtual hands directly scanned from the real world have been integrated, after a retopology process, in a virtual environment created for the Metaverse, and have been integrated with digital nails. Human interaction with the Metaverse has been carried out through the acquisition of the real posture of the user’s hands using an infrared-based sensor and mapping it in its virtualized version, achieving natural identification. The technique has been successfully tested in an immersive shopping experience with the Meta Quest 2 headset as a pilot project, where a transactions mechanism based on the blockchain technology (non-fungible tokens, NFTs) has allowed for the development of a feasible solution for massive audiences. The consumers’ reactions were extremely positive, with a total of 250 in-person participants and 120 remote accesses to the Metaverse. Very interesting technical guidelines are raised in this project, the resolution of which may be useful for future implementations.

## 1. Introduction

The constant development of the Internet of Things (IoT) together with the possibilities of creating cyberspaces has been leading to a convergence between the physical and virtual world [1]. In recent times, particularly after the COVID-19 pandemic, human activities like work, shopping, and entertainment have been moving to online forms. Today, people spend more time in virtual spaces. New commercial forms enable more industries to seek innovative development methods, especially in pioneering realms like electronic games, fashion, or education [2].

In this general context, in recent times, the term Metaverse has expanded and popularized. Although it was originally introduced in 1992 by the American novelist Neal Stephenson in his science fiction book “Snow Crash” to refer to a version of the internet [3], the concept of the Metaverse is not yet fully defined. Instead, it is evolving as the Metaverse itself is created.

What we can say is that the word Metaverse is a portmanteau composed of the prefix “meta” (which in Greek means “beyond” or “after”) and “verse” (which refers to the “universe”). That is, a universe beyond what we know, making use of 3D elements that can be interacted with to generate immersion [4]. But, as opposite to what one might think, the Metaverse is not only for playing games, but also a digital world with persistence and synchronization, as well as economy, culture, regulation, ethics, and morality. Therefore, it must be equipped with advanced techniques to keep activities, interactions, and transactions safe, transparent, and sustainable [5].

A critical point that marked the arrival of the Metaverse to the general public was the presentation by Mark Zuckerberg, CEO and founder of Meta, in October 2021. In this talk, Zuckerberg assured that the Metaverse would be available in 5 or 10 years and could be accessed from virtual reality (VR) and augmented reality (AR) devices. Meta is working on the creation of an open Metaverse, which will join the rest of this company’s platforms [6].

Currently, there is a wide variety of companies working on developing platforms for the Metaverse around the world. Some examples are: Roblox, Fornite, Decentraland, Sandbox, Somnium Space, Virtway, Shiba…

The aspects that are being worked on and characterize the Metaverse are the following:

- Dynamic environments in which the user can interact with objects in the space or with other users;
- The combination of digital twins with the creation of totally unique spaces that do not exist in reality;
- Respect for the laws of real-world physics;
- Both centralized or decentralized architectures, based on the platform’s control element (centralized Metaverse is managed by a single company or person, while the decentralized Metaverse is controlled by all users);
- The incorporation of virtual economies, through the implementation of a buying and selling mechanism based on blockchain technology, as happens with non-fungible tokens (NFTs);
- The use of customizable avatars to make the user feel identified with their alter ego;
- Different access devices, such as VR or AR glasses, as well as conventional devices such as a smartphones, tablets, or PCs.

To fulfill the need for an immersive user experience in the Metaverse, it is necessary to carry out innovative research. In [7], the authors established that the Metaverse is not a composition of one or more technologies, but it depends on six technical pillars named BIGANT: Blockchain, Interactivity, Game, Artificial Intelligence, Network, and IoT. In [1], the authors concluded that there are four pillars of the Metaverse:

- Ubiquitous connections;
- Space convergence;
- Virtuality and reality interaction;
- Human-centered communication.

These pillars make it possible to break physical boundaries and temporal limitations and achieve an immersive user experience in the Metaverse. 

In this rapidly changing environment, it is critical that a multitude of initiatives emerge aimed at exploring both technology and the forms of human interaction with this new framework. 

Precisely in this area is where the present project is framed, which presents itself as a case study of a specific problem in the fashion field in which the inclusion of the Metaverse provides added value and allows for the exploration of new possibilities of interaction, communication, and expression.

The contribution of this work is threefold. First, a pioneer application is presented where shopping is moved to an online and VR form in an innovative way in the realm of fashion [8]. Second, a viable technical solution is proposed to bring VR fashion nails to a massive audience through the combined use of WebXR, the OpenSea marketplace, and the MetaMask crypto wallet. Third, an immersive shopping experience is provided to those owning VR devices with a 3D dynamic environment to interact with, through the implementation of a buying and selling mechanism based on blockchain technology, NFTs. This solution is equipped with advanced techniques able to keep activities, interactions, and transactions safe, transparent, and sustainable.

This paper is organized as follows. In Section 2, the related work is introduced, both referring to other fields of applications, and to technical developments that can be applied in the specific problem addressed in this study. Section 3 introduces the project in which the present work has been developed. In Section 4, a description of the different devices used in the project along with the software is detailed. Section 5 details the development itself, the decisions taken in the process, as well as the different aspects of its implementation. Section 6 discusses the results of the project. Section 7 contains a discussion about the results, and some future developments are proposed. Finally, Section 8 is a conclusion of all the presented information.

## 2. Related Work

The emergence of the Metaverse is having a dramatic impact on a wide range of applications with a direct and notable effect on human daily life. In recent times, we have witnessed initiatives aimed at introducing the new paradigm in the most unexpected sectors. In [9], the authors provide an interesting discussion on technologies for virtual reality simulation games. In [10], similar technologies are used for the reconstruction of historical and industrial heritage for cultural enrichment. 

In [11], an interesting proposal is described to bring the metaverse to the field of tourist services and cultural promotion, while in [12], a deep reflection on the interrelation between the metaverse and the world of digital art is detailed. More particularly, [13] is an outstanding proposal on holographic models applied to the design and exhibition of architectural spaces. 

The problem of interacting in the Metaverse without the need to use dedicated hardware is acquiring great relevance in recent times [14]. The use of specific peripherals makes the management of characters in virtual environments less intuitive, which makes the implementation of new technologies difficult. This is the reason why there is considerable research in order to find the right way for the user to have the feeling of being immersed in a synthetic environment. And one of the key points in this activity is the need to capture the movement of the hands to route it to the user’s virtual twin.

Wearable resistive sensors that could directly characterize joint movements are a promising technology for hand gesture recognition due to their easy integration, low cost, and simple signal acquisition. In [15], the authors summarize the common categories of wearable resistive sensors for hand gesture recognition and review the recent advances in the field.

A considerable effort was made in marker-based optical hand tracking, sometimes referred to as ground truth tracking, which requires individual markers to be placed on each tracked finger/hand and a complicated network of specialized and carefully calibrated optical or magnetic sensors [16,17].

As opposed to marker-based tracking, optical markerless motion-tracking technology has become one of the most cost-effective alternatives for tracking hand and finger movements across a wide range of applications [18]. 

The use of markerless motion tracking is spreading rapidly amongst researchers from a wide range of scientific fields due to the relatively small number of hardware components involved and reduced costs. Only as an example, in the robotics field, accurate human hand and finger tracking data are used to safely teach robots to grasp various objects and to perform complex interactions in a simulation environment before being deployed in the real world [19]. In the field of human–computer interaction, it allows for research into more natural ways of interacting with virtual objects in immersive environments [20]. In psychology and neuroscience, it has the potential to enable research into a wide variety of areas, such as tool use, social interactions, and rehabilitation in VR [21].

## 3. The Project

Ana González Rodriguez is a fashion designer and creative director of the brand Ana Locking, founded in Madrid in 2008 [22]. During her time as a judge on the program “Drag Race Spain”, she wore a collection of nails from her brand. After seeing the reception they had on Twitter, she wanted to take them further. To achieve this, it was proposed to digitize and include them in the Metaverse, and thus give them greater visibility.

That idea led her to contact the company MetricSalad, with experience in implementing events in the Metaverse. The innovative nature of the proposal, together with the fact that it involved developments and resources that were not yet available to the company, made it advisable to involve different departments of the university, the research history of which could nourish the project and supply the materials and techniques necessary for its final achievement. The project also entailed an important design restriction: it had to be finished for public exhibition in a massive event for the press and the public. Many of the decisions were made taking into account the time constraints [23].

In carrying out this project, other objectives have been addressed such as research on VR, AR, XR, metaverses, and NFT; business model proposals with the purpose of making this type of project profitable; solutions that bring VR to as many people as possible to change the vision they have of it and demonstrate that there are applications beyond the world of video games.

## 4. Materials and Methods

In this section, we will describe the selected materials to develop the project. 

### 4.1. Hand Tracking

Hand tracking is a form of technology that captures hand movement, in order to map it onto a virtual hand or manipulate virtual objects. After decades of research in artificial intelligence applied to image processing, it has been possible to replicate the complexity of hand movements, and this allows for the positioning and rotation of the user’s fingers to be recognized in real time. This offers the opportunity for a more realistic experience to the users, thus enhancing the immersion in virtual reality.

**Figure 1:**
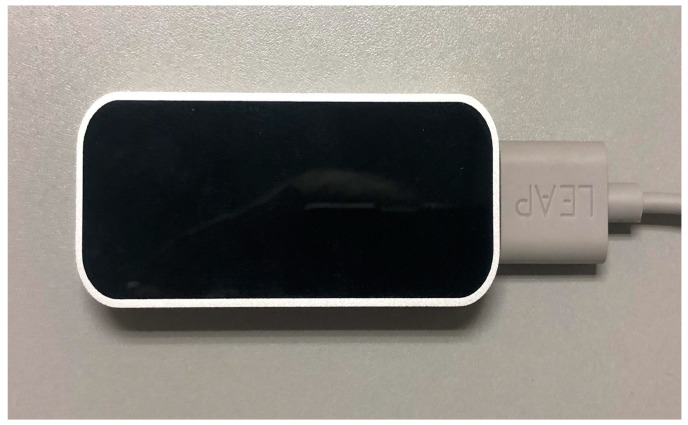
Leap Motion Controller device.

**Figure 2:**
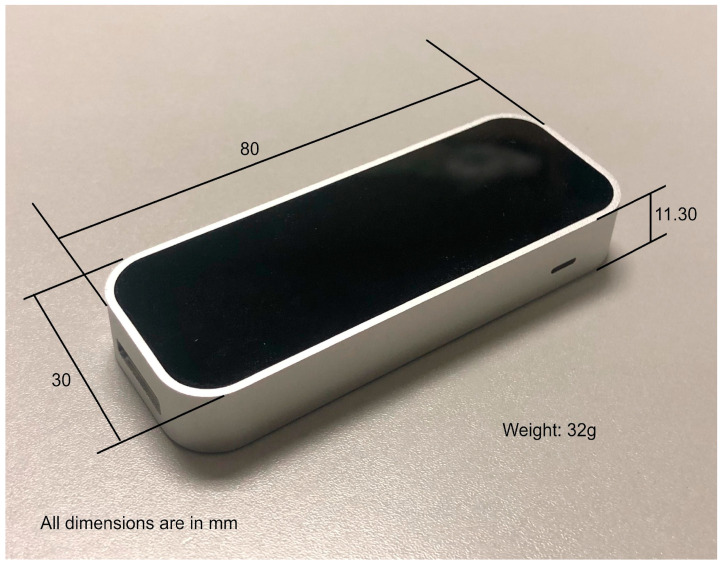
Leap Motion dimensions and weight.

Although there are some solutions for hand tracking that make no use of dedicated peripherals, such as Manomotion library [24], performance tests lead us to rely on the Leap Motion Controller, a specific device used for obtaining data on hand movement in real time that can be seen in Figure 1. The Leap Motion Controller makes interacting with digital content natural and effortless [25]. It is a small and light element, as shown in Figure 2, made up of two 640 × 240 pixel infrared cameras. This allows it to capture hand movement in an area of 60 cm or more, extending from the device in a typical field of view of 140 × 120°. It detects both hands, all the fingers, and even the user’s forearm.

To do this, the Leap Motion Controller incorporates LEDs that illuminate the hands using infrared lights, invisible to the human eye. The cameras have a motion latency below the human threshold of perception. Thanks to this, a really fluid result is achieved.

The Leap Motion Controller can distinguish up to 26 different points on each hand, even if they are hidden behind others, at least theoretically. However, too much overlapping in a movement can cause tracking problems in real time, as experienced through the development of the project.

The first version of the Leap Motion Controller arrived to market in 2012, but it was not until 2015 when the first version aimed at integrating with VR headsets was released. With the VR Developer Mount accessory, the user can connect the device with a VR headset easily and safely. Consequently, the controller has three basic modes of operation:

- Desktop, face up;
- Desktop, face down;
- Mounted on a VR headset.

There are different plugins to use Leap Motion in software environments such as Unity 2023 or Unreal 5.

### 4.2. Meta Quest 2

**Figure 3:**
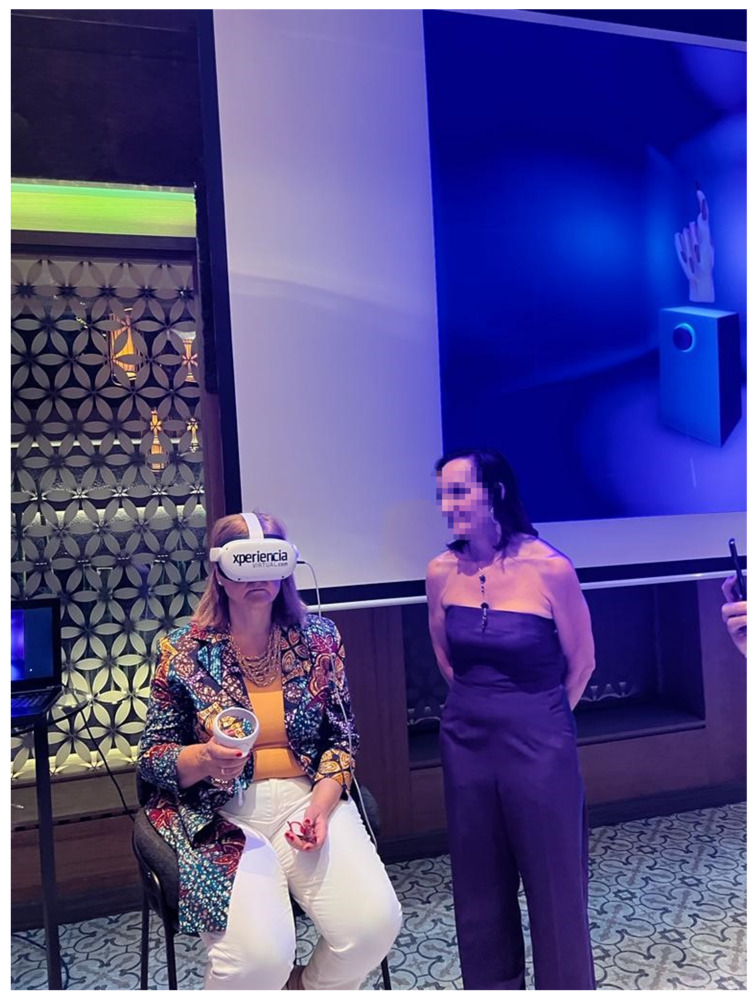
Meta Quest 2 device being used in demonstration event [23].

Quest 2 is the model of VR headset that has been used to develop, test, and demonstrate the project. This device was selected due to its versatility and high compatibility with VR programs [26]. It can be seen in Figure 3.

VR headsets consist of a helmet with two screens inside. The existence of these two screens manage to trick the user’s brain by offering a slightly different image to each eye, creating the impression of the visualization of a 3D scene [27]. 

The headset comes with two controllers, which allow the user to interact with the space, but they are aimed to people with a certain background in VR or 3D applications. For the general public, they can be somewhat counterintuitive and difficult to adapt to. The headset includes a sophisticated 3D sound system, but they also have a jack in case the user wants to connect higher quality headphones, a peripheral that can significantly improve the experience.

Four position cameras are included on the front of the headset. These cameras are used for detecting the headset position in the room; that way, the software can track the movement of the user and map it onto the virtual world, thus warning if the user has moved too far and there is a risk of collision. The guardian system takes control of the safety zone, which is configured every time the headset is turned on. 

The lenses on the headset have different positions, and they can be adjusted depending on the distance between user’s eyes. The lenses route the images from two LCD panels.

Meta Quest 2 is considerably lighter than previous releases, namely Oculus Quest, a fact that improves user experience. It also can be run without having to be connected to a computer or a battery, unlike the previous model, Oculus Rift.

It is worth noting that first experiences with a VR headset like the one selected can provoke a little dizziness and disorientation, a fact that is being exhaustively studied to improve the user experience [28].

### 4.3. Anet Handy Sense

**Figure 4:**
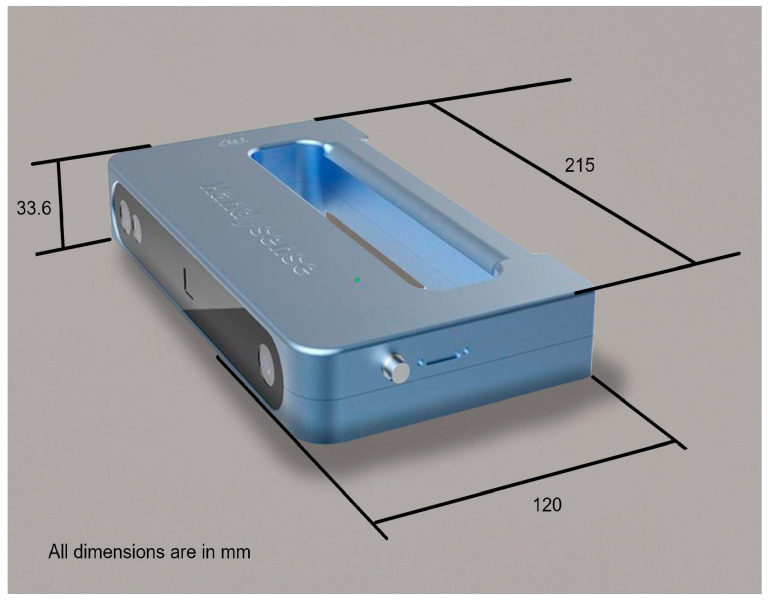
Anet Handy Sense device with dimensions.

Anet Handy Sense is a handheld scanner with a 3D camera, with a compact and lightweight design, which allows the user to work in coordination with a laptop [29]. The device is shown in Figure 4.

It uses dual-chamber infrared structured light technology, which is safe for skin and eyes. It is focused on usability, with minimum intervention from the user. The device works at a speed of 10 fps, and allows for the export of the results of the scanning process in different formats such as obj, stl, 3mf, asc, or ply.

### 4.4. Software

Several software tools have been used during the development of the project. In this subsection, we present only those with the greatest impact on the final result.

#### 4.4.1. Blender

Blender 4.0 is a free and open source software, which can create 3D visualizations as well as still images, 3D animations, and VFX shots [30].

It has become popular due to the combination of several factors: intuitive use that helps beginners perform simple tasks such as changing the position, size, and orientation of an object; and the extensive additional technical complexity in terms of animations, UV maps, shaders, meshes, and modifiers. Another great advantage of this software is the compatibility with other creation programs and video game engines, such as Unreal Engine or Unity.

Although, initially, it was conceived as a commercial tool, in 2002, Blender was released as open source under a GNU license.

#### 4.4.2. Unity

Unity is a cross-platform video game graphics engine [31]. It was first released in June 2005 at Apple Worldwide Developers Conference as a Mac OS X game engine. Since then, the engine has been gradually extended to support a variety of desktop, mobile, console, and virtual reality platforms. It is particularly popular for iOS and Android mobile game development. It is easy to use for beginners, and very popular in indie environments.

The engine can be used to create three-dimensional (3D) and two-dimensional (2D) games, as well as interactive simulations and other experiences. The engine has been adopted by industries such as film, automotives, architecture, engineering, and construction. 

#### 4.4.3. Spark AR

Spark AR v174 is a free-to-use application for creating AR effects for Instagram, Facebook, and Messenger. Users and companies can create, publish, and share effects [32].

Spark AR offers a wide variety of options to customize designs. It allows for the import of 3D objects, audio files, and script packages. In addition, it offers the Scripting option that allows for the addition of logic and interactivity beyond the default options, using the JavaScript programming language. With this, it is possible to animate static objects, create textures and materials, modify/distort a facial mask, interact with elements, and many other options. The software includes a tracking function capable of detecting a body, a hand, or a face.

#### 4.4.4. OpenSea

OpenSea 7.0 is a decentralized marketplace focused on the purchase and sale of digital assets (NFTs) based on blockchain technology [33]. The platform is responsible for managing smart contracts on blockchain networks such as Ethereum, Polygon, and Solana. It has become popular thanks to its simple and easy-to-use interface, exceeding 500,000 users and 40 million registered NFTs.

#### 4.4.5. MetaMask

MetaMask 11.7 is an Ethereum crypto wallet for storing tokens and Non-Fungible Tokens (NFTs) [34]. It is an active wallet, which means that it needs to be run on a device connected to the internet. MetaMask allows the user to have different accounts, each with a different address, in the same crypto wallet.

MetaMast is distributed in the form of a mobile app or web browser extension, operating on browser such as Chrome or Firefox. MetaMask is developed by ConsenSys Software Inc., a blockchain software company focusing on Ethereum-based tools and infrastructure.

### 4.5. Hardware and Software Requirements

The devices and programs referred to imply a series of practical restrictions on the development system, which are detailed below.

Among all the software tools, the one that implies the most requirements is the use of Unity 2022, which is summarized in Table 1.

If the system is capable of running Unity, then it can also easily run Blender, as well as the Leap Motion control software v5.16, which takes the form of a Unity package.

Regarding the XR platform, for it to run correctly on the Meta Quest 2, the headset must have software version 39 or later installed.

Spark AR, OpenSea, and MetaMask are web applications that run in a browser and do not impose additional restrictions on the system.

For guidance purposes only, all the developments were carried out on Intel Core i7-6700K CPU 4.00GHz Dell computers, sourced from Valencia, Spain, with 32 GB of RAM, running Windows 10 Enterprise. Additionally, to speed up the graphics processing, the computers contained NVIDIA GeForce GTX 970 Graphic Processing Units (GPUs).

## 5. Development

This section describes all the stages of project development, with special emphasis on how the necessary resources were managed to obtain the desired result.

### 5.1. Virtual Room

**Figure 5:**
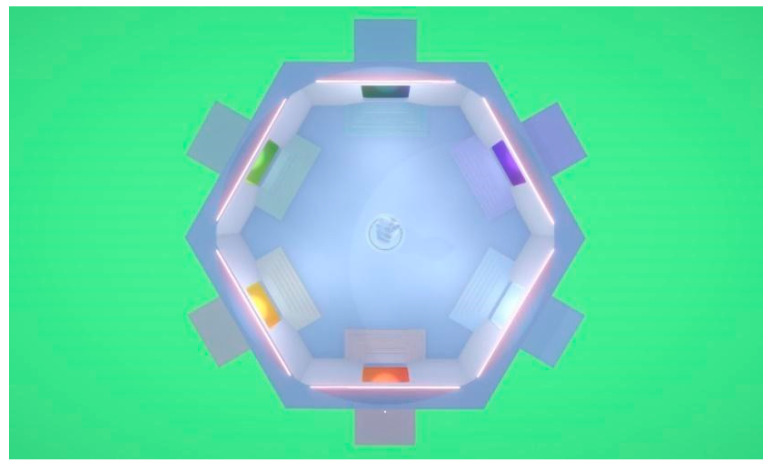
VR room. Top view.

**Figure 6:**
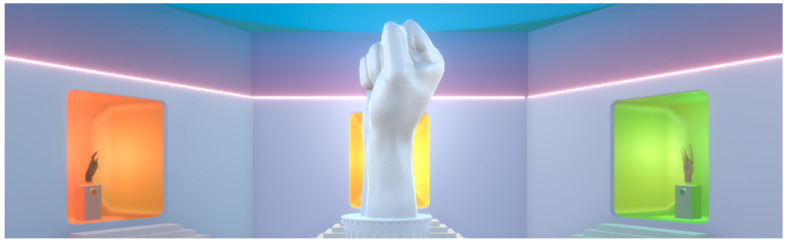
VR room. Front view.

As a first step in the development of the project, it was necessary to model a room to house the application in the virtual environment. For this, Blender was used, following some reference images. The results can be seen in Figure 5 and Figure 6.

Once the model is available, it is taken to Unity where the interactivity will be programmed.

To support virtual reality, we use Web XR, a format that combines the possibility of viewing the same project on a web server from the computer or from a VR or AR device, such as the Meta Quest 2 VR glasses.

To configure WebXR, it is necessary to use the Oculus XR plugin package, which provides input and display support for Oculus devices and offers support for the built-in editor for OpenXR.

It will also be necessary to use the Mixed Reality Toolkit (MRTK) library, a Microsoft project that includes a set of features and configurations to facilitate and accelerate the development of VR applications [35]. MRTK incorporates spatial and user interface (UI) interactions and offers different versions compatible with a wide range of platforms.

In this application, it is crucial that the environment connects with an external browser to launch third-party applications. For instance, it is necessary to communicate the virtual room with the Open Sea marketplace where the NFTs of the nails are sold. This need implies the use of the OpenWindows.jslib plugin, which must be added to each object with which it is going to interact. The actions will be controlled by the MRTK PressableRoundButton prefab, supporting different user inputs. When selecting events, care will be taken to respect the immersiveness of the environment.

### 5.2. Virtual Hands

The hand model was obtained through 3D scanning using the HandySense 3D Scanner device from Anet manufacturer, sourced from Valencia, Spain. The scanner was configured to obtain a mesh with a resolution of 1.5 mm, which gives enough detail to reflect accurately the geometry of a hand. The scanning process proved to be quite noisy, and was not without problems.

Since it is an animated object, the solution of keeping the scanner stationary and using a turntable to visualize all the geometry is not feasible. Instead, it is the scanner itself that has to rotate around the hand, which must also be kept as still as possible throughout the entire process, which lasted several minutes. For this purpose, the use of mechanical supports can help.

The other important factor to control during the process is lighting. In the fixed scanner and turntable configuration, the light source remains fixed and is not affected by the scanner. But when it is, the scanner that has to move around the object; this does not happen, and spurious shadows may appear. The solution to this problem is to add several complementary light sources and use diffused light for general lighting. 

**Figure 7:**
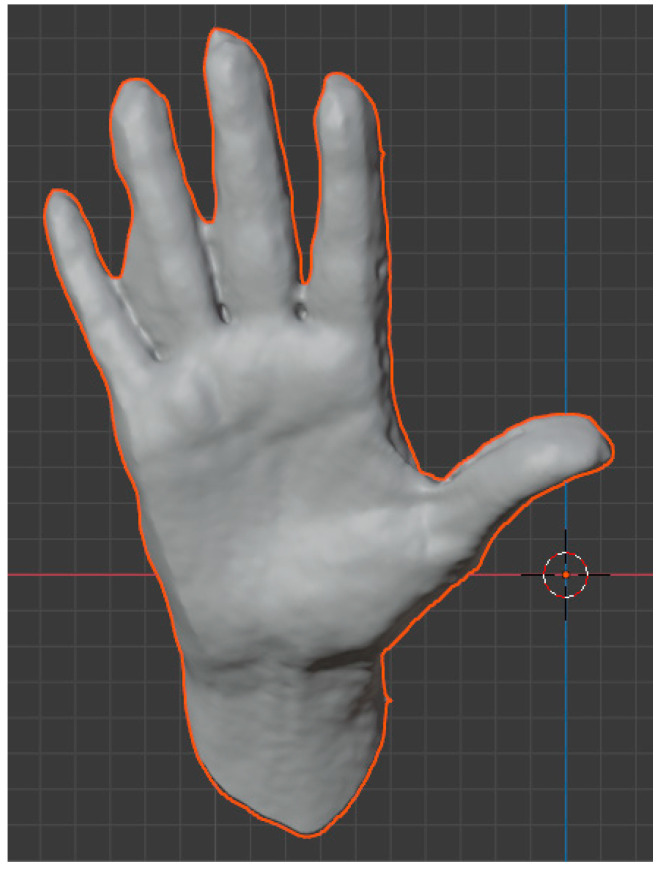
Raw scanned hand model.

As a result from the scanning process, a cloud of points is collected, which are joined into a mesh, to form the 3D model, composed of 69,209 vertices, joined into 138,414 triangular faces. Scanning is not a perfect process, and a lot of imperfections were obtained, as can be seen in Figure 7.

**Figure 8:**
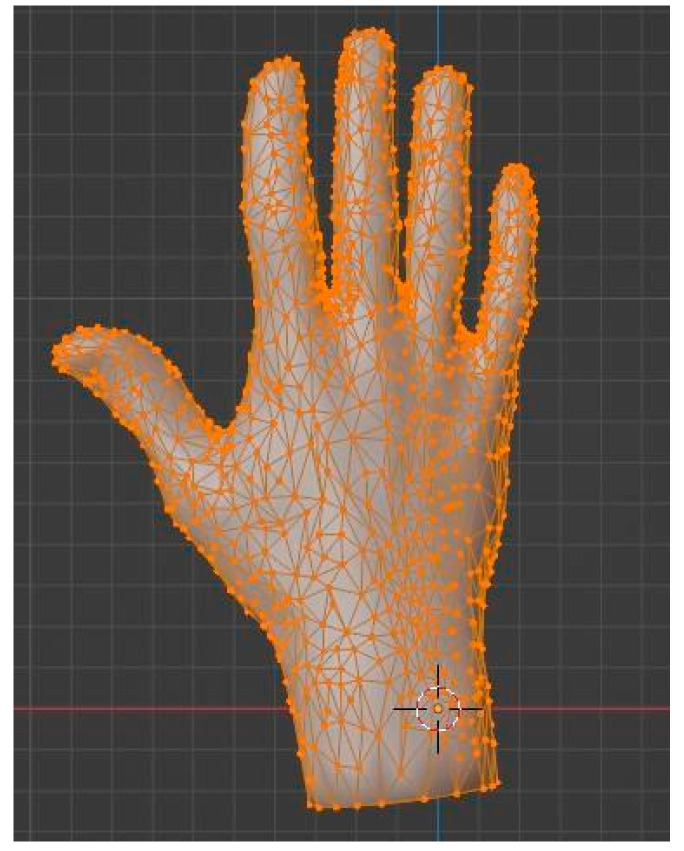
Hand model after clean-up and decimation.

As it is evident from the image, there are parts of the model where information is missing, with holes in the mesh, or where little movements have induced some noise. This is the reason why the model must be cleaned by eliminating noise points and filling in the holes. Also, the number of points should be reduced for the model to be easily handled in the software. This process is called decimation, and blender offers several tools to perform it with a great accuracy and reduction rate. The new hand was composed of 1703 vertices joined in 3402 triangular faces, as can be seen in Figure 8.

The geometric model obtained from the scan represents reality in sufficient detail, but it is not suitable when the model must be animated, since the topology of the mesh, a priori, does not coincide with the deformation characteristics of the object. In the case of the hand, this constitutes an important limitation, and it is necessary to introduce a retopology process, which leads to a topologically correct model that follows the 3D distribution of the original mesh.

**Figure 9:**
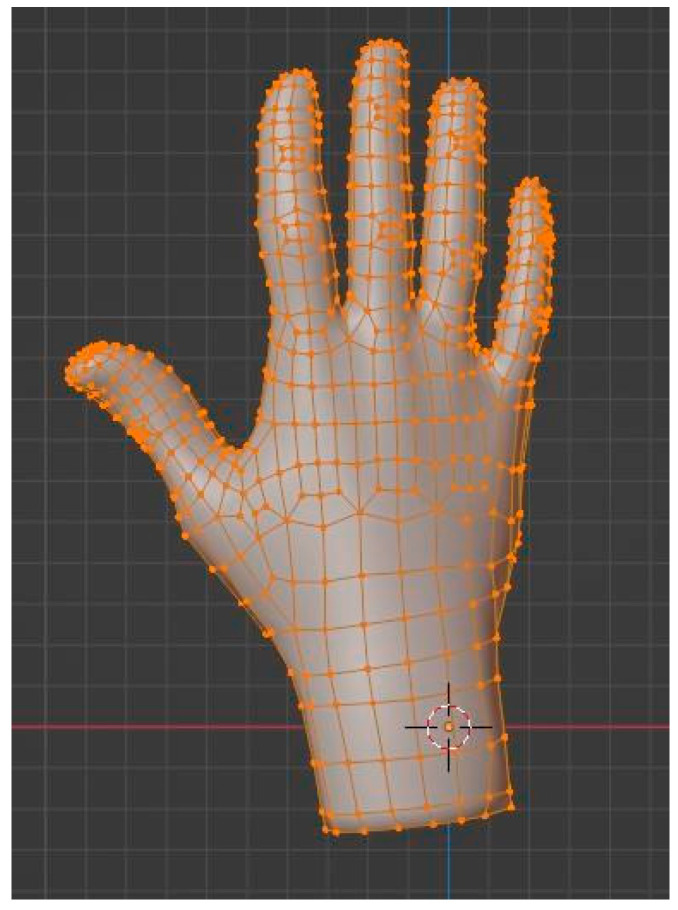
Hand model after retopology.

Although some programs offer resources for automatic retopology, if the target is to perform an animation, the correct option is to carry out a manual procedure, in which the designer places the polygons one by one. For placement, the logical lines formed by the joints of the phalanges must be followed, enclosing each of them in a loop of vertices. Once this is completed, the number of cuts is increased, filling the inter-articular space with vertices for which such proximity is no longer necessary. Figure 9 can serve as a guide to check the placement of the loops. The result of this slow but crucial process is a model made up of 1003 vertices joined into 1006 faces. The important thing about these faces is that they are no longer triangular, but quadratic, which gives them better characteristics for deformation and animation. 

**Figure 10:**
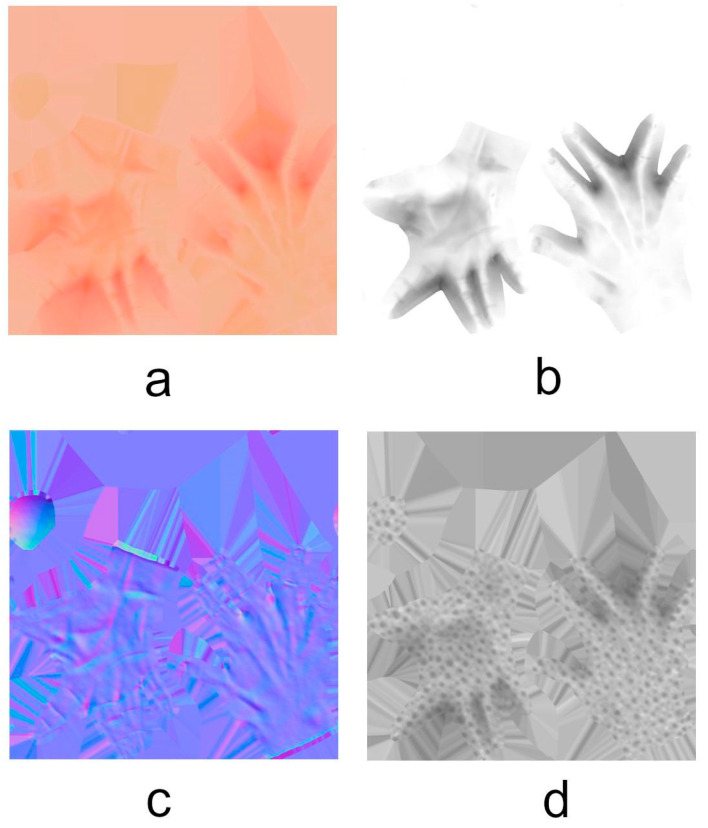
Texture files to map the detail of the original hand to the optimized one: (**a**) base color, (**b**) ambient occlusion, (**c**) normal map, and (**d**) roughness.

At first, it would seem that a model with only 1000 vertices is unable to capture the detail of the original, with more than 50,000. However, there are techniques to translate the detail from the original to the mesh after decimation and retopology. In our case, we made use of multiresolution to bake the original mesh detail into four texture files that will then be applied to the optimized model. These files contained, respectively, the base color, ambient occlusion, normal map, and roughness of the model. The textures are presented in Figure 10. In this way, the visual detail of a model with low geometric complexity is increased.

After this, the model is prepared to undergo a rigging process, which consists of creating a structure of bones and movement points within the geometry to match the joints of the hand.

The support structure for rigging is the Armature, made up of Bones. Each Bone has three different parts: Root, Body, and Tail.

Once obtained, it is necessary to link the Armature and mesh through a parenting process, so that the deformations of the Armature affect the spatial position of the points of the mesh with the correct influence.

### 5.3. Virtual Animation

The animation of the hands is not the result of design but is acquired from the movement of a real hand through corresponding sensorization. For this task, Leap Motion hand tracking software is used. The SDK includes the interface between Unity and the Ultraleap hand tracking software. It enables the physical representation of hands and controls in virtual reality scenes including grasping, throwing, collision feedback, and proximity detection functions. Moreover, a set of scripts allows for the linking of the device data with the 3D models.

Models can be extended with the appropriate hierarchical structure. For instance, in this project, the designed nails can be attached to the hand by making them sons of the last finger bone. In a logic way, the nail must be placed at the last point of the corresponding finger, so that it is in the right position, but without affecting the animation process. It is just an anchor point, with no joint movement. 

**Figure 11:**
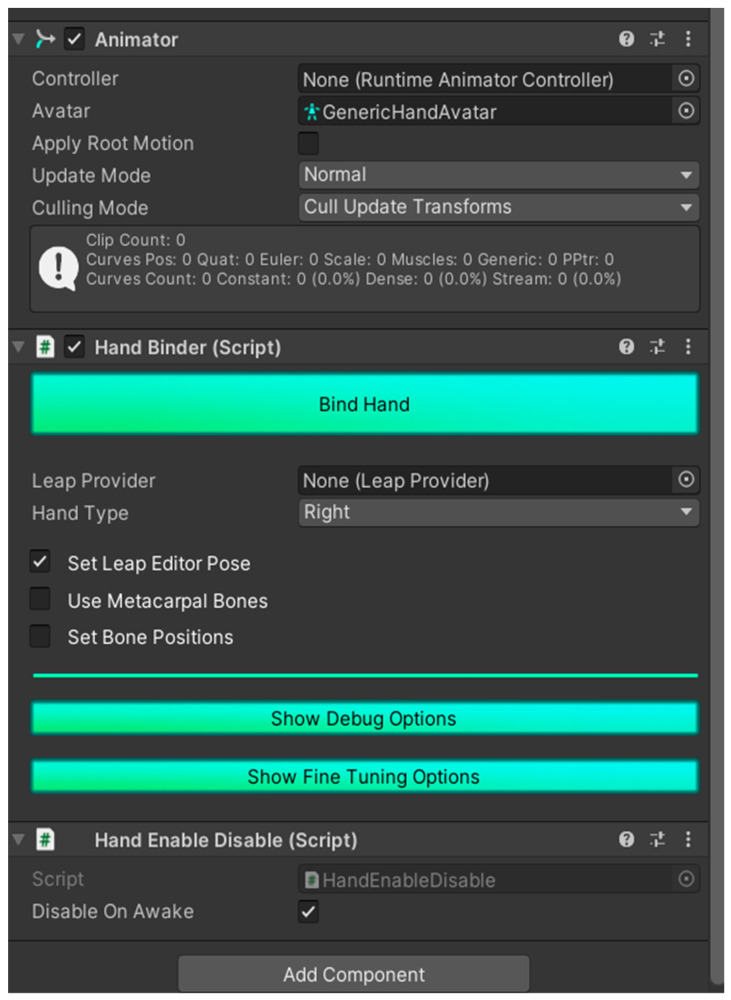
Complete setup of a Unity object to animate the hand with Leap Motion.

Three components must be added to the hand model:

- The Hand Binder script allows one to link the 3D model with the Leap Motion software;
- The Hand Enable Disable script is used to configure the correct operation of the Hand Binder script, as it avoids alerts due to collisions with itself;
- The Animator allows one to play previously recorded hand motions recordings.
- The complete setup is shown in Figure 11.

The Leap Motion application allows for the tracking of hand movement in real time. If the nails have been successfully attached to the tip of the fingers, they move accordingly.

There is also the possibility of recording the movement in the form of an animation that can be reproduced afterwards. In order to do this, the Unity Recorder package must be installed.

**Figure 12:**
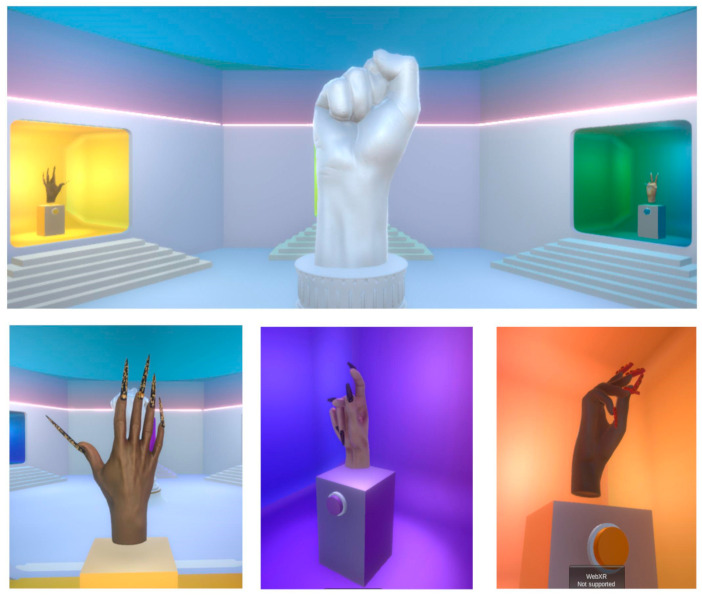
Hand and nails placed in the virtual room.

The virtual room is stored in a web page [36]. The result can be seen in Figure 12.

### 5.4. NFT Creation

Tokens are units used in a business model and to which a value is assigned. Non-Fungible Tokens are not consumed when used, and they cannot be replaced by another token, since they are unique. The value of an NFT is based on the digital asset that guarantees its originality, uniqueness, and irreplaceability. Many of them are actually pieces of digital art minted using blockchain technology, which develop secure and reliable traceability with all the information of their creation and copyright.

The operation of NFTs is defined under the blockchain technology network, just like cryptocurrencies. For each NFT, a smart contract, a digital certificate of authenticity, is created, which includes a set of metadata to guarantee it. It records the starting value, information on acquisitions or transactions, as well as the author’s information. In this way, the value assigned to the NFT in each of the transactions and the addresses of each of the owners are recorded.

For the process of creating an NFT in Open Sea, a Wallet must be connected. In this case, MetaMask has been chosen as it is the most popular option. MetaMask facilitates interaction with applications on the Ethereum blockchain and allows for the storing of digital items.

After the entire configuration process, the supplier can upload the resource to the sales website, where it will be available. It is also possible to manage the exchange process, setting the price and publishing market offers.

### 5.5. Instagram Filter

To accompany the launch of the virtual experience, making use of the resources generated, and to create expectation regarding the product, an augmented reality filter was developed for Instagram. For this target, Spark AR Studio was used.

This filter tries to detect the movement of the face of the user. For this, a Face Mesh and a Face Tracker component are needed. A Face Finder is added that indicates the different faces that the camera finds. Next, it is linked to a Face Select node to indicate which face is selected from those indicated by the previous node. That way it is possible to track the movement of one face. 

With all this information, it is possible to add a 3D object (the hand with the nails). Due to the inherent limitation of 4 MB from Spark AR, different filters were developed for each model of the nails in the collection.

The animation starts when the user opens the mouth in a WOW expression. When the event triggers, the hand with the nails appears, and it reproduces an animation obtained with the VR application. The filter has been published in Spark AR Hub, so it can be used by other users from Instagram.

## 6. Results

**Figure 13:**
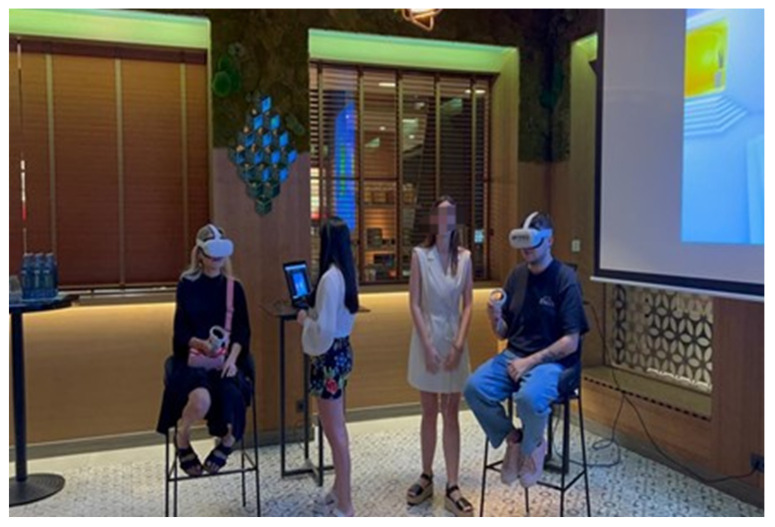
A photograph of users in the demonstration event.

The public presentation of the project took place at a massive event in Madrid [23]. Journalists and different influential people in this field were invited to attend this event. A photograph of this event can be seen in Figure 13.

In order to bring the possibility of viewing the room from VR glasses to more people and as an advertising strategy, the central point of the event was an immersive experience in which attendants used the Meta Quest 2 headset to view the room. To guide the experience, external screens were installed that allowed access to the user view through the “Share screen—Stream” option. This way, it was possible to upload video to a computer connected to the same Wi-Fi network as the headset. 

The reactions of the consumers were impressive. Although the technology is not fully developed and still has a long way to go, the experience was seen as realistic, as well as innovative. We appreciated the so-called “wow effect” in the public exposed to VR for the first time. This effect was reinforced with the artistic design of the room, which was colorful and LGBT+ oriented. 

Nevertheless, to achieve a complete VR experience, it is necessary to add some interactivity. The priority in this case was to carry out this process in an elegant way, moving away from the idea of an animated video game that some people have when talking about VR. To do this, the interactive elements that have been added are the skybox that depends on the time, the hand animations, and the buttons that link us to the OpenSea website. The fact that there are moving objects, hands, in the room helps improve the experience, allowing the user to feel more immersed in virtual reality.

A total of 250 participants had the opportunity to test the system on-site, a high number, taking into account that each experience took an average of 5–10 min, so that the user had time to become familiar with the environment. In addition to the tests at the site itself, 120 external accesses to the metaverse were recorded from users connecting remotely. As a result, in the days following the event, there were around 50 press articles in different national media, most of them with very positive reviews about the great possibilities of metaverse technology [37,38]. However, it must be said that there were some critical media, although the objections can normally be attributed more to a certain skepticism and issues of an economic nature, such as the possibility of speculation. [39].

Regarding the collection of NFTs, it was launched as a test of market research. The objective was to find out the best way to carry out this process in future projects and analyze the public’s response to that market niche. The result helped analyze the features of the process, taking into account that no marketing plan was developed to advertise the sale. In fact, the published press releases focused more on the virtual world than on the sale of these NFTs. 

However, it was possible to see that among the two main blockchains to create an NFT, Ethereum and Polygon, Ethereum is the most popular and where most of the NFTs released to the market are produced. This is the reason why there is a greater number of collections in it and the best-known artists are found there. When carrying out these transactions, gas rates are paid, which are usually very high due to network congestion. Although this is inconvenient, it also helps control the network so there is less spam. It offers the function of auctioning NFTs, something that is not available on Polygon.

Polygon is a protocol built to connect Ethereum-compatible blockchain networks; it emerged as a second-layer solution to relieve Ethereum congestion. It uses side chains, which makes it faster. The gas rates are much lower, even zero in some cases, so it offers us a more economical solution. 

For our project, Polygon was considered the best option, because its costs are reduced and, in addition, it is less harmful to the environment. This is due to its marketing plan, based on sustainability and reducing the carbon footprint. 

During the event, five NFTs were sold. This is not a high figure, but we once again emphasize the fact that the event was conceived as a demonstration, rather than a sales event itself.

One of the critical points when presenting the Metaverse to the general public is the evaluation of the accuracy with which the posture of the hand is sensed.

In our project, since it was a demonstration event to disseminate the technology to the public, the use of Leap Motion was chosen because, in specifications, it ensured greater accuracy than other alternative resources.

It should be said that, at runtime, some accuracy and acquisition problems were experienced. However, during the demonstration, it was difficult to assess whether the problems were inherent to the device or, on the contrary, came from incorrect handling by the user. We did note, among other things, that those users with some prior experience in VR environments reported fewer errors than those who came with no prior experience. It must be considered, for example, that the correct acquisition of the hand posture implies that they must always remain within the field of vision of the device, which may not be immediate at first.

Nevertheless, given, on the one hand, the informative nature of the project and, on the other hand, the short development deadlines established, a detailed evaluation of the accuracy was left out of the present study and is pending in future extensions.

## 7. Discussion and Future Work

The results detailed above indicate the success of the initiative regarding the implementation of a viable immersive VR shopping experience in the Metaverse, developed for massive audiences. The boundaries of the currently available technology have been explored and some insights can be drawn from this project, as well as their limitations. In this sense, the problems or barriers overcome by the project can serve to develop similar initiatives in other fields.

First, very few HMIs today can offer an immersive experience to users. In fact, only VR/AR glasses are available at a reasonable price and even these are still expensive (from USD 800 to USD 3000). It can be foreseen that, in the near future, new devices will arise that will provide a global sensory experience and not just a visual one, as has been available up till now. There exist other devices appropriate for human interaction with virtual environments, such as instrumented gloves, motion capture suits, or eye-tracking glasses, but the price and complexity of their use make them unattainable for non-specialized users.

Second, the VR shopping experiences must be developed in safe environments, especially if the user can move around the setting, as occurs in a shop. In this regard, the development of AR experiences instead of VR experiences can provide a more realistic and secure experience to the user, due to the merging of a real environment with virtual objects to interact with. However, at present, there are very few glasses that offer the possibility of mixing reality and virtuality.

Although the project met the specifications initially proposed, these had to be limited to objectives that could be addressed in the time available for the development, which left out some features that could have been interesting and that, over time, should be included:

- Broadcast streaming videos in real time using WebXR;
- Offer the experience in multiplayer mode, so that users can interact with each other. This fact implies using avatars, and text and voice chat;
- Introduce a geolocated AR experience, which varies the content displayed depending on the geographic position of the user;
- Increase the degree of interactivity, using all the possibilities offered by MRTK2.

Apart from these characteristics, it must be taken into account that the sector is evolving rapidly, and it is necessary to make a significant effort to keep up to date. In that sense, two of the critical devices used in the project have already been replaced for more modern versions. During the development time, Meta Quest 3 and Ultraleap 2 have entered the market. Any new development in the presented sense should abandon the old devices and replace them with the new, more sophisticated ones.

Finally, it is important to note that the technology is still in an early stage. Above all, it should be noted that, currently, there is no single Metaverse concept, but rather different developers are building their own metaverses, with the dispersion that this implies. It would be expected that, just as the Internet once found a consensus structure to which all networked applications adapted, the same will happen with the Metaverse, which will acquire a unique form with which different users and developers will interact, each one with its specific objective. In the future, there may be a single Metaverse that we can use to hold immersive virtual meetings, work, carry out economic transactions, receive medical or psychological therapy, play immersive games, or perform virtual tourism. While this is happening, it is interesting to see how different companies are making a strenuous effort to implement their devices, particularly HMIs like those used in this project. The final form that this Metaverse will take will depend on how these devices evolve to be within reach of the general public. 

## 8. Conclusions

In this paper, a case study has been shown of a specific problem in which the inclusion of the Metaverse provides added value and allows for the exploration of new possibilities of interaction, communication, and expression.

The problem addressed falls within the field of fashion and consisted of the implementation of a Metaverse in which the user can purchase virtual nails to be attached to a virtual hand that reflects the user’s movements in the real world.

Time restrictions were crucial for the development of the project, given that it was conceived as a technological demonstrator to disseminate new trends among the general public. This fact conditioned the use of low-cost devices with moderate specifications.

During the public exhibition, widespread interest in innovative initiatives was noted that contributed to disseminating the possibilities that the Metaverse can offer to daily life.

Although the technology is still in an early stage, the authors defend the importance of these events so that our developments permeate society.

## Figures and Tables

**Table 1:** System requirements for Unity 2022 Editor.

Requirements	Windows	macOS	Linux
Operating system version	Windows 7 (SP1+), Windows 10 and Windows 11, 64-bit versions only.	Mojave 10.14+ (Intel editor) Big Sur 11.0 (Apple silicon Editor)	Ubuntu 20.04 and Ubuntu 18.04.
CPU	X64 architecture with SSE2 instruction set support	X64 architecture with SSE2 instruction set support (Intel processors) Apple M1 or above (Apple silicon-based processors)	X64 architecture with SSE2 instruction set support
Graphics API	DX10, DX11, and DX12-capable GPUs	Metal-capable Intel and AMD GPUs	OpenGL 3.2+ or Vulkan-capable, Nvidia and AMD GPUs.
Additional requirements	Hardware vendor officially supported drivers	Apple officially supported drivers (Intel processor) Rosetta 2 is required for Apple silicon devices running on either Apple silicon or Intel versions of the Unity Editor.	Gnome desktop environment running on top of X11 windowing system, Nvidia official proprietary graphics driver or AMD Mesa graphics driver. Other configuration and user environment as provided stock with the supported distribution (Kernel, Compositor, etc.)

## Data Availability

Data are contained within the article.
